# Self-Neutralizing Melamine–Urea–Formaldehyde–Citric Acid Resins for Wood Panel Adhesives

**DOI:** 10.3390/polym16131819

**Published:** 2024-06-27

**Authors:** Xuedong Xi, Antonio Pizzi, Hong Lei, Xiaojian Zhou, Guanben Du

**Affiliations:** 1 Yunnan Key Laboratory of Wood Adhesives and Glued Products, College of Material Science and Engineering, Southwest Forestry University, Kunming 650224, China; xuedong.xi@swfu.edu.cn (X.X.); xiaojian.zhou@swfu.edy.cn (X.Z.); guanben.du@swfu.edu.cn (G.D.); 2 LERMAB-ENSTIB, University of Lorraine, 88000 Epinal, France; 3 College of Chemistry and Material Engineering, Zhejiang A&F University, Hangzhou 311300, China; leihong@zafu.eud.cn

**Keywords:** wood adhesives, MUF resin, citric acid, buffer, self-neutralizing acid system, organic base–organic acid buffer

## Abstract

In this study, we used a self-neutralizing system to counteract too acidic a pH, unsuitable for wood adhesives, and tested it on MUF resins augmented by the addition of citric acid or other organic acids, based on the addition of small percentages of hexamine or another suitable organic base to form an acid–base buffer. In this manner, the pH of the adhesive was maintained above the minimum allowed value of 4, and the strength results of wood particleboard and plywood bonded with this adhesive system increased due to the additional cross-linking imparted by the citric acid. Thus, the wood constituents at the wood/adhesive interface were not damaged/degraded by too low a pH, thus avoiding longer-term service failure of the bonded joints. The addition of the buffering system increased the strength of the bondline in both the plywood and particleboard, both when dry and after hot water and boiling water tests. The IB strength of the particleboard was then increased by 15–17% when dry but by 82% after boiling. For the plywood, the shear strengths when dry and after 3 h in hot water at 63 °C were, respectively, 37% and 90% higher than for the control. The improvement in the bonded panel strength is ascribed to multiple reasons: (i) the slower, more regular cross-linking rate due to the action of the buffer; (ii) the shift in the polycondensation–degradation equilibrium to the left induced by the higher pH and the long-term stability of the organic buffer; (iii) the additional cross-linking by citric acid of some of the MUF resin amine groups; (iv) the already known direct linking of citric acid with the carbohydrates and lignin constituents at the interface of the wood substrate; and (v) the likely covalent linking to the interfacial wood constituents of the prelinked MUF–citric acid resin by some of the unreacted citric acid carboxyl groups.

## 1. Introduction

In the last few years, considerable research has been conducted on citric acid used either as a direct wood binder [1,2,3]; as an active cross-linker monomer of different biomaterials such as glucose, starch or other carbohydrates [4,5,6,7,8,9,10]; or as a contributing factor in the upgrading of both other bioadhesives [11,12] and synthetic adhesives [13]. The results obtained have been very promising using all these research approaches, to the point that research has been extended to the use of even stronger acids such as p-toluenesulfonic acid coupled with biosourced materials such as glucose [14]. Again, the results have been promising. However, a very fundamental problem has not been considered, and only recently has this problem come to the fore, namely the strong acidity of all these adhesives at the wood/acid interface with a pH that is very low. Unfortunately, while the polymerization of citric and other acids with glucose and other carbohydrates seems clear, such an adhesive system cannot be used for wood, as most global wood bonding standards state that too acidic an adhesive, with a pH lower than 4, cannot be used on wood as it would degrade it by hydrolyzing the polymeric wood constituents at the adhesive/wood interface [15,16,17,18,19]. Several industrial cases of joint failures due to this reason are known in the world. In the majority of papers where the pHs of acid–carbohydrate adhesives are cited [5,14,20,21,22], the pHs tabled are far too low, between 0.8 and 2, to be acceptable for wood bonding. Very recently, an effective system for the self-neutralization of adhesives based on citric acid and carbohydrates has been presented [23] based on an old system used to self-neutralize acid-setting PF resins [24]. While this approach has worked for starch–citric acid adhesives, it does not solve the problem of upgrading traditional adhesives through a partial co-reaction with citric acid or other acids. Thus, a new and simpler system suitable for contributing to upgrading traditional adhesives needs to be developed. The present research work addresses this drawback for melamine–urea–formaldehyde resins as an example of a traditional synthetic wood adhesive.

Melamine–urea–formaldehyde (MUF) adhesives are one of the main thermosetting wood adhesives used to bond a variety of wood panels. Several systems have been reported both for upgrading their performance as well as to decrease the proportion of melamine [25,26,27,28,29,30,31]. Since the original research group discovered the effect of citric acid [1], several authors have shown the potential of this natural product to bind particleboards using several types of biomass [2,3,4,5,6], to bond wide flat wood surfaces such as LVL and plywood [3] and to markedly improve the water resistance of wood-welded joints [11].

Recent work has shown that the upgrading by citric acid of MUF adhesives occurs both with the resin and with wood lignin and the carbohydrate constituents of wood [13]. This idea was based on the disponibility of alcohol groups in UF and MUF resins and PF resols, in the form of the hydroxymethyl groups abundant in such resins, to react through esterification with citric acid during curing of the adhesive. Among these thermosetting resins, the one chosen for reaction with citric acid was MUF, because such a resin presents a more complex system in which a number of different reactions may occur. The reactions foreseen to occur are esterification between the carboxylic acid functions of citric acid and both the adhesive amino groups and the numerous aromatic and aliphatic hydroxyl groups of the main wood constituents [3]. The acid reaction with the lignocellulosic substrate at both the level of the wood polymeric carbohydrates and lignin also contributes to the bonding improvement [11].

For many years, research has shown that MUF resins in the presence of a small percentage of an organic base does form a buffer when an acid is added, thus slowing down the decrease in pH without really affecting the reactivity and performance of the resin [31]. This system was originally conceived to markedly reduce the proportion of expensive melamine in the MUF resin while conserving the adhesive’s exterior performance [30,31]. It has already been used industrially for some niche applications [32]. The present research work presents the application of this system to self-neutralize MUF–citric acid resins to be able to safely take advantage of the upgrading by citric acid without causing any damage to the wood substrate. The novelty of the work presented here is in rendering possible the use of a reinforcing system of adhesives through the use of citric acid, a biosourced material, without its acidity damaging the wood at its interface with the adhesive, without which such an improvement as reinforcement with citric acid cannot be used.

## 2. Materials and Methods

### 2.1. MUF Resin and Glue Mix Preparation

The melamine–urea–formaldehyde (MUF) resin was prepared in the laboratory with the following procedure: the pH of 300 g of a 37% formalin/water solution was adjusted to 9, and then, the first loads of urea (74 g) and melamine (20 g) (all three obtained from Sigma Aldrich, Saint Louis, Missouri, USA) were added and the temperature increased to 90 °C. Upon 90 °C being reached, the pH was adjusted to 5–5.5 and maintained as such for 40 min. The pH was then adjusted to 8.5 and the second load of melamine (63 g) added, and the reaction was maintained for 60 min. The temperature was then decreased to 60 °C and the pH adjusted to 8, followed by the addition of the second load of urea (19.59 g). The reaction was then maintained as such for a further 20 min and then cooled in ice and kept in the refrigerator up to the time of being used.

### 2.2. Titration and Buffer Action

The titration of the resins was carried out using 20 g resin with a 60% resin solid content, and this was titrated with citric acid (Sigma Aldrich, Saint Louis, MO, USA) t a 30% concentration in water or with p-toluenesulfonic acid (Sigma Aldrich, Saint Louis, MO, USA) at a 10% concentration in water. The results are given in the figures in actual milliliters of these two solutions needed to reach a given pH upon adding 2% or 3.3% of hexamethylene tetramine (hexamine) (Sigma Aldrich, Saint Louis, MO, USA) by weight to the total weight of MUF resin solids as a buffer counterbase.

### 2.3. Cross-Polarization–Magic Angle Spinning–Nuclear Magnetic Resonance (CP-MAS ^13^C NMR) Spectra

The MUF resin + 20% by weight of citric acid was hardened in the oven at 103 °C for 2 h and then crushed to a very fine powder. The citric acid + MUF hardened resin was analyzed by CP MAS 13C NMR. A Brüker Avance (Brüker France, Wissembourg, France) 400 MHz spectrometer was used at a carbon resonance frequency of 100.6 MHz to obtain the spectra. A 4-microsecond impulse duration at 90° was used. The rotor was spun at 12 kHz on a double-bearing 4 mm Bruker probe. A 1 ms cross polarization contact time was used with a 100% ramp with continuous wave decoupling. Between 4000 and 10,000 scans were used.

### 2.4. MALDI-TOF Analysis

The MUF resin + 20% by weight of citric acid was hardened in the oven at 110 °C. The samples were then treated with a NaCl solution (1.5 µL of 0.1 M) in a methanol/water mixture (1:1) to increase ion formation, and placed on the MALDI target and dried. The samples and the matrix were then mixed in equal amounts, and 1.5 µL of the resulting slurry was placed on the MALDI target and dried at 40 °C for 2 h before being analyzed. A matrix of 2,5-dihydroxy benzoic acid was used. Red phosphorous (500–3000 Da) was used as reference for spectrum calibration. Finally, after evaporation of the solvent, the MALDI target was introduced into the spectrometer.

The spectra were recorded on a KRATOS AXIMA Performance mass spectrometer from Shimadzu Biotech (Kratos Analytical Shimadzu Europe Ltd., Manchester, UK). The irradiation source was a pulsed nitrogen laser with a wavelength of 337 nm. The length of one laser pulse was 3 ns. Measurements were carried out using the following conditions: polarity—positive; flight path—linear; 20 kV acceleration voltage; 100–150 pulses per spectrum. The delayed-extraction technique was used, applying delay times of 200–800 ns. The software MALDI-MS (AXIMA Performance) was used for data treatment. The oligomers could appear in the spectra either corresponding to their molecular weight or to their molecular weight +23 Da of the Na+ ion derived from the NaCl used as an enhancer. The spectral precision was +1 Da.

### 2.5. Particleboard and Plywood Preparation and Testing

Duplicate one-layer laboratory particleboard with dimensions of 350 × 310 × 14 mm was then produced from an industrial wood chip furnish composed of 70% by weight of beech and 30% by weight of spruce by adding 10% total MUF resin solid content to the dry wood particles together with 20% by weight of citric acid plus 3% hexamine to the total MUF resin solids, pressed at a maximum pressure of 28 kg/cm^2^ (2 min from platen contact to high pressure + maintenance of high pressure) followed by a descending pressing cycle of 2.5 min at 12–14 kg/cm^2^ and 3 min at 5–7 kg/cm^2^, at 190 °C, for a total pressing time of 7.5 min. The resinated chips’ moisture content was 12%. The target density of the panel was 690–700 g/cm^3^. The same pressing conditions and resin loads were used for an MUF control with the addition of a 3% ammonium sufate hardener to resin solids and with a glue mix formed of MUF resin + 2% p-toluenesulfonic acid and 3.3% hexamine.

Triplicate three-ply laboratory plywood panels of 450 mm × 300 mm × 5 mm were prepared using 2.3 mm thick poplar (*Populus tremuloides*) veneers. To the MUF resin, we added 20% by weight of citric acid plus 3% hexamine to the total MUF resin. A 260 g/m^2^ double glue line was the glue mix load applied to the veneers. The plywood was pressed for 8 min at 150 °C at a pressure of 1.5 MPa. The plywood panels were then conditioned for 48 h at 20 °C and 2% relative humidity. The panels were then tested according to China National Standard GB/T 14074 (2006) [33] and China National Standard GB/T17657 (1999) [34], which require a minimum average shear strength of 0.7 MPa on five specimens tested, and European Norm EN 636:2012 (2012) [35].

## 3. Results and Discussion

**Figure 1:**
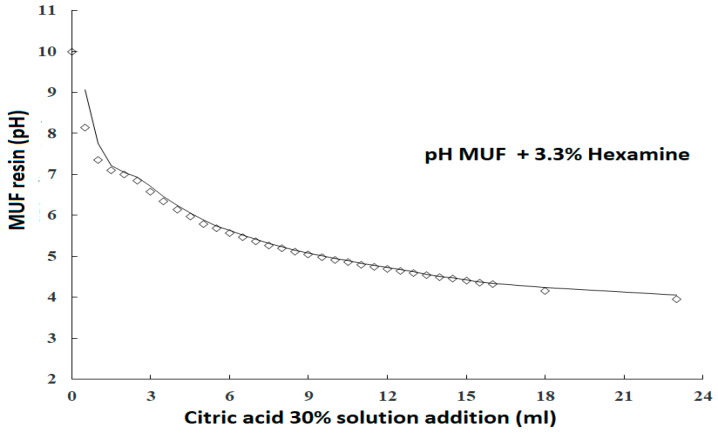
Variation in pH as a function of titration with 30% citric acid solution of a 20 g MUF resin with a 60% concentration, and of the same MUF resin + hexamine.

**Figure 2:**
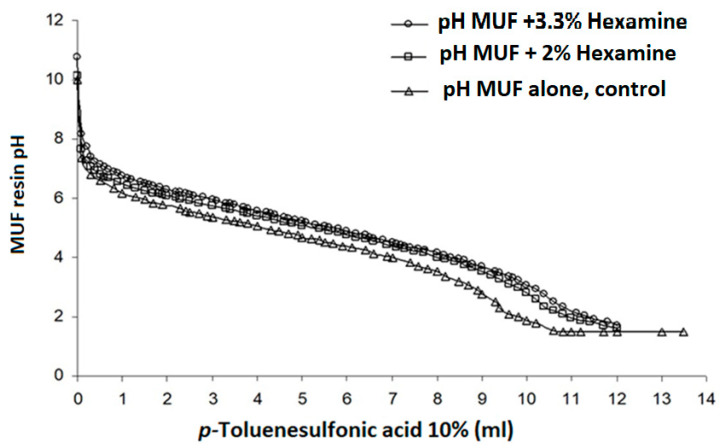
Variation in pH as a function of titration with 10% p-toluenesulfonic acid solution of 20 g of MUF resin with a 60% concentration, and of the same MUF resin + hexamine.

Hexamine was the base used in this experiment to form a hexamine–citrate buffer and a hexamine–p-toluenesulfonic acid buffer [31]. Some other organic bases can also be used for this purpose, such as morpholine [29]. The low amount of hexamine does not lead to a formaldehyde increase, first because the proportion is too small, and second as under the conditions used, hexamine is well documented not to produce formaldehyde but reactive aminomethylene bases [29,30,31,36,37]. The trends indicated in Figure 1 for the system MUF–hexamine–citric acid and in Figure 2 for the system MUF–hexamine–p-toluene sulfonic acid indicate that effect of buffering is well present in both systems; this is closely linked to the buffer action that the resin and the base–acid salt or complex exhibit during curing. The data for the effect of 3.3% hexamine on resin solids in the MUF–citric acid adhesive system for an M/U molar ratio of 47:53 and an F/(M+U) molar ratio of 1.5 are shown in Figure 1.

To reach a pH of 4, up to 24 mL of a 30% citric acid solution in water is needed, and thus, 30% solid citric acid on the MUF resin solid weight/weight. The buffer effect is then very marked and would allow the use of relatively high proportions of citric acid without any potential degradation of the wood surface constituents.

The trend is similar for the proportions of 2% and 3.3% hexamine in the MUF-p-toluenesulfonic acid adhesive system for the same resin molar ratios. Thus, in Figure 2, to reach pH 4, around 8 mL of 10% p-toluenesulfonic acid, and thus, 4% acid solids in the MUF solid content (weight/weight), is needed, even when just 2–3.3% hexamine is present (Figure 2). Conversely, for the same concentration of p-toluene sulfonic acid ions on the pure resin without hexamine, a pH of 4 is reached with just 2.5% acid solids in the MUF solids (wt/wt) (Figure 2).

In both cases, once hexamine is present, the reaction should be sufficiently fast but still relatively slower than without hexamine, yielding a better strength result without damaging the lignocellulosic/adhesive interface as a consequence of the enhanced buffering effect. This can only be observed with 10% p-toluenesulfonic acid. These results show that buffer effect should be as marked as possible, but equally that the buffer effect must be limited to maintain the resin within a relatively narrow and well-defined range of pH. If the pH of stabilization of the titration curve, and, hence, of buffering, is too high, the curing of the resin will be too slow. This would have disastrous results when using industrial short curing times. If the pH of stabilization of the titration curve, and, hence, of buffering, is too low, resin curing would still be too fast to obtain much improved strength, with equally disastrous results.

The conclusion is that any molecules able to maintain the buffer effect for long enough within a certain narrow pH range should have the same effect as has the hexamine–acid system. However, the effect is more complex. Regarding the buffering effects already used for MUFs for other purposes, it has already been shown that, for example, the use of ammonia as a base also provides buffering, but it also gives titration curves leading to too high a pH range. The buffering is thus too marked, and thus, the too-high pH obtained precludes the formation of a hardened resin network of reasonable performance. Thus, buffering occurs but is also outside of the pH limits within which reasonably good bonding strength can be achieved.

### 3.1. CP MAS ^13^C NMR

**Figure 3:**
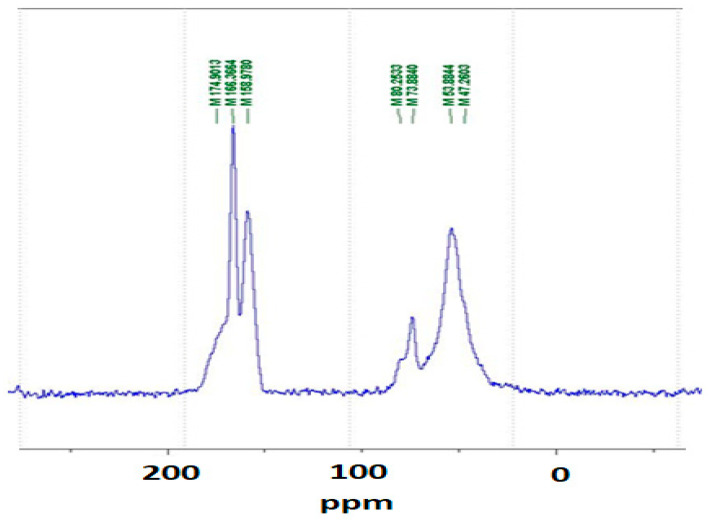
The 13C NMR of the MUF + citric acid resin.

The CP MAS ^13^C NMR of the MUF + citric acid in Figure 3 yields no more information that what is generally shown in a polymerized MUF resin. Thus, the broad 174–175 ppm peak is characteristic of unreacted and monosubstituted triazine. The 166 ppm peak is assigned to the signals of tri-substituted and multi-substituted triazine and of unreacted urea. Disubstituted and trisubstituted urea are shown by the 158–159 ppm peak.

The small 80 ppm shoulder is characteristic of the free formaldehyde in the MUF adhesive, while the peak at 73–74 ppm belongs to branched methylene ether bridges between the melamine amine and/or urea amide groups. The -CH_2_OH group on hydroxymethylated melamine or urea shows up as a 53–54 ppm peak. The conclusion is that the NMR spectrum is not able to discern the role of citric acid in the hardened MUF resin.

### 3.2. MALDI ToF

**Figure 4:**
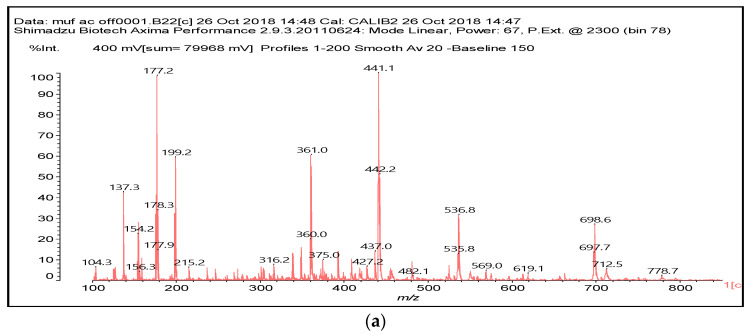

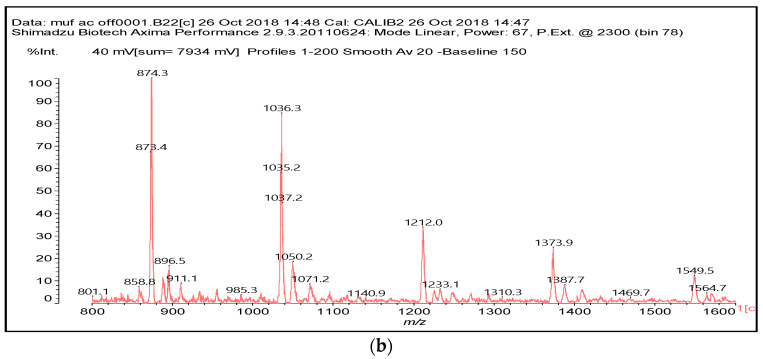
MALDI ToF spectra of MUF resin + 20% citric acid in the (**a**) 100 Da–800 Da range and (**b**) 800 Da–1000 Da range.

**Figure 5:**
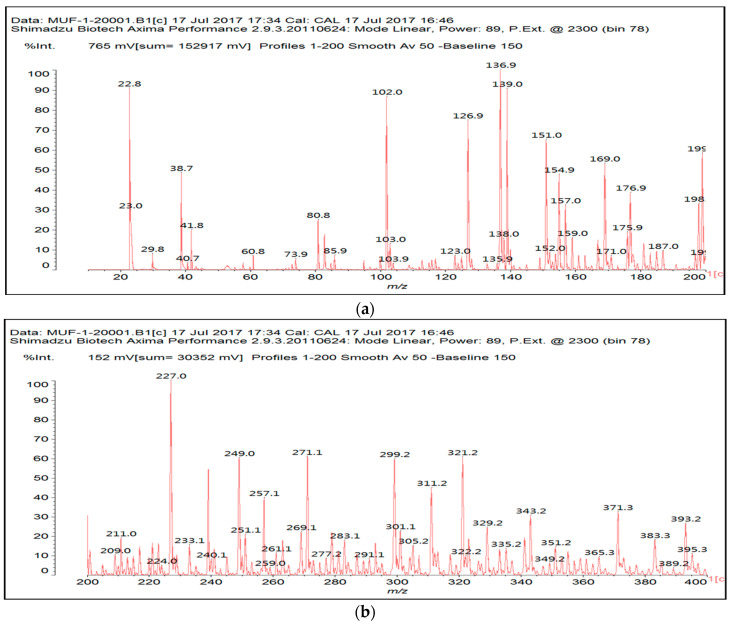

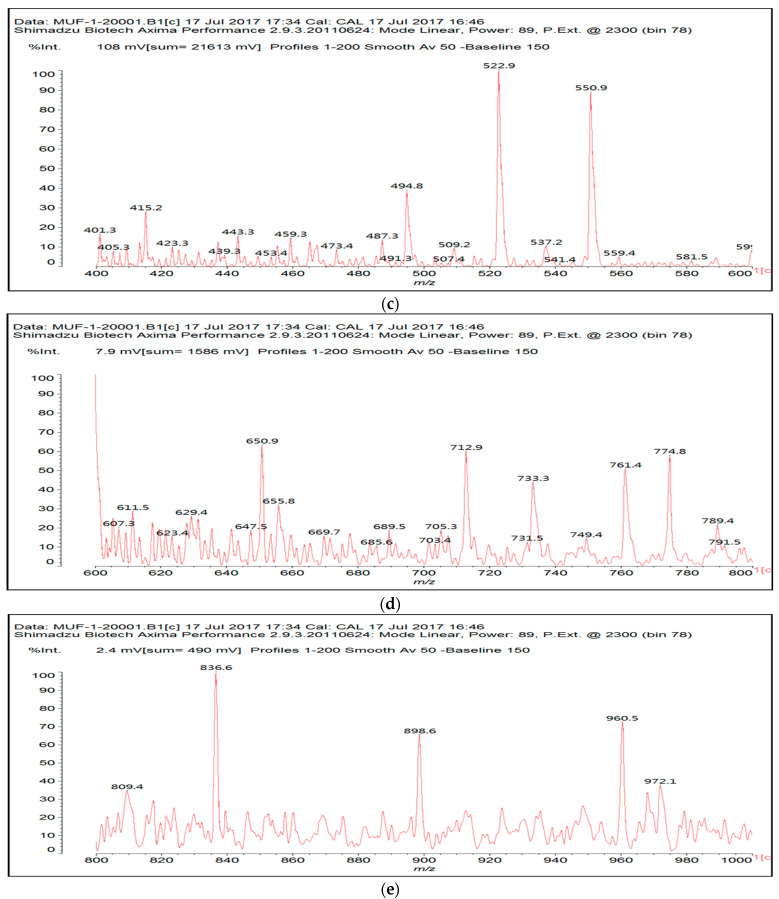

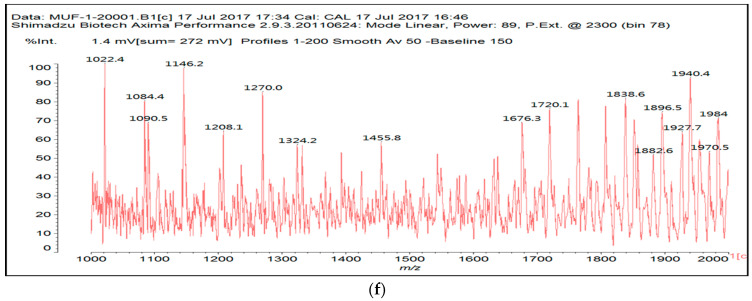
MALDI ToF spectra of MUF resin alone in the (**a**) 20 Da–200 Da range, (**b**) 200 Da–400 Da range, (**c**) 400 Da–600 Da range, (**d**) 600 Da–800 Da range, (**e**) 800 Da–1000 Da range, and (**f**) 1000 Da–2000 Da range.

The MUF control resin MALDI ToF analysis revealed the formation of reaction compounds by the polycondensation of melamine and urea with formaldehyde, as would be expected (Table 1, Figure 4 for MUF control and Figure 5a–f for MUF + citric acid). The reaction of citric acid with the MUF resin instead yields a number of different compounds according to the MALDI ToF analysis. Starting from 199 Da, a peak series increasing by 162 Da and 175/176 Da in alternate steps occurs with peaks at 199 Da–361 Da–536 Da–698 Da–874 Da–1036 Da–1212 Da–1373 Da–1549 Da, etc.

This might appear as an alternate sequential addition of two different residues. One explanation may be that one of these is a monoreacted citric acid (MW = 175 Da), and another explanation may be that it is a –O-CH_2_-U-CH_2_-U residue (MW = 162 Da). Some of these products are also formed, but they are only formed in a limited proportion, indicating that the explanation above is true in part only. This is because the –O-CH_2_-U-CH_2_-U reaction possibilities are rapidly exhausted if there is no further reaction with formaldehyde. However, this is unlikely, as then, the repeating motive would be greater than 162 Da due to the extra –CH2- group added. However, the possibility of this occurring does exist as a sequential alternation of –O-CH_2_-U-CH_2_-U and –O-CH_2_-U-CH_2_-U-CH_2_^+^ residues reacted with formaldehyde either still free in the resin or released by its methylene ether bridges. The direct reaction of urea’s –NH_2_ amide groups with a carboxylic acid is not possible, thus rapidly limiting this polymerization route to condensation to species of lower molecular weight.

The melamine case is different, as its –NH_2_ groups are aminic (not amidic). Thus, amine groups of melamine can react with carboxylic acid groups, but only at relatively higher temperatures than ambient, to yield –CO-NH- amide groups of the carboxylic acid used. Consequently, citric acid –COOH groups can react with (i) melamine –NH_2_ amine groups forming amide bridges and (ii) the hydroxymethyl groups (-CH_2_OH) formed by reaction of formaldehyde with melamine and urea, yielding a –COOCH_2_-NH- ester bridge. The formation of both these structures has already been shown, indicating that both of the reactions indicated do occur with ease, and the totality of the species formed have already been determined and reported [9]. A clear example is structure I determined by MALDI ToF, which includes citric acid esterification of the –CH_2_OH hydroxymethyl groups of a UF oligomer and bridges generated by its reaction with melamine amine groups.


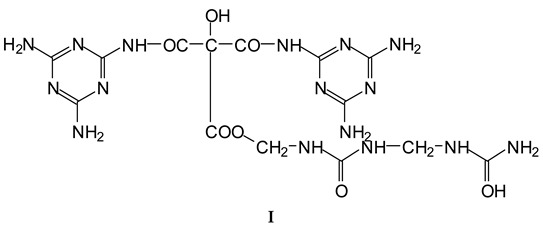


Longer oligomers are also generated by such a complex set of reactions, yielding, for example, structure II, also determined by MALDI ToF, where two citric acid residues have reacted.


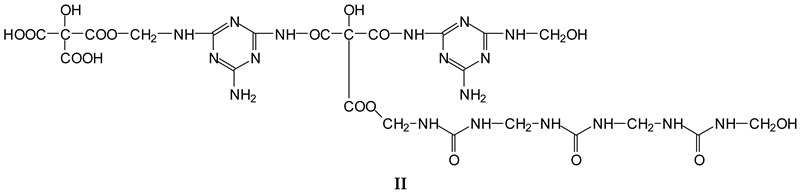


We can summarize the set of reactions occurring as follows:

(1). -COOH + H_2_N- = -CONH- + H_2_O;
(2). -COOH + HOH_2_C- = -COOH_2_C- + H_2_O;
(3). 2 R-NH_2_ + HCHO = R-NH-CH_2_OH = R-NH-CH_2_-NH-R’.

As melamine can react only at high temperatures with the acid, the resin was hardened at 103 °C for 2 h to determine the full range of compounds that would be formed [9]. Plywood panels are generally bonded at a high veneer interface temperature of 120 °C, but only for a few minutes, inferring that ester bridges may predominate. Conversely, for panels where the press temperature is traditionally much higher, such as particleboard, LVL, and OSB, with temperatures between 180 °C and 220 °C, the formation of amide bridges is rendered possible too by the direct reaction of melamine.

To conclude, the system MUF + 20% citric acid is composed of a very complex set of reactions. Thus, superimposed onto the normal MUF oligomers formed by the reaction with formaldehyde, ester bridges and amide bridges are formed, the latter exclusively with melamine, and the former with the hydroxymethyl groups on either urea or melamine [13]. It appears that the esters are formed through preference on the urea-carrying hydroxymethyl groups rather than on those on the melamine [13], although their presence cannot be excluded.

Lastly, the reactions of citric acid with the wood surface constituents are superimposed onto the above reactions, reactions that have already been detailed [3].

### 3.3. Particleboard and Plywood Test Results

The results of the three-layer plywood panels prepared after hot pressing for 8 min at 150 °C are shown in Table 2. The results clearly indicate that the shear strength of the panels with 30% citric acid both when tested dry and when tested after the 2 h boiling test is higher than that of the MUF control. Namely the MUF + citric acid yielded average shear strengths when dry and after boiling, respectively, 38% and 90% higher than the MUF control.

The results in Table 2 for the laboratory particleboard panels show a similar trend to those for plywood, with both the neutralized citric acid and p-toluenesulfonic acid glue mixes. The self-neutralized MUF + citric acid adhesive yielded average internal bond (IB) strengths when dry and after boiling, respectively, 14% and 88% higher than the MUF control, while the self-neutralized MUF + p-toluenesulfonic acid adhesive yielded average internal bond (IB) strengths when dry and after boiling, respectively 16% and 82% higher than the MUF control.

These results indicate that the addition of citric acid or even of a stronger acid, such as p-toluene sulfonic acid, to a thermosetting MUF wood adhesive improves both the dry strength and, to an even greater extent, the boil strength of the bonded joint with a glue mix, the minimum pH of which is not lower than 4, or higher depending on the proportion of acid used; this means that the adhesive is then perfectly acceptable for wood bonding.

There are a number of different reasons for such improvement in the bonding results with self-neutralized acids systems.

The reasons initially considered to cause such effects are as follows:

(i). The slower cross-linking due to the buffer action certainly plays some role, but it is not the main cause of the improved performance, as has already been ascertained before [29].
(ii). Previous work has determined that the main factor is that the polycondensation–degradation equilibrium is shifted to the left due to the higher pH [31], but it is limited to a narrow buffering pH range. At a much lower pH, degradation predominates, and at a much higher pH, polycondensation becomes too slow to be of use.
(iii). A third contributing factor is the stability of the organic base–acid buffer; hence, the buffer stability is of a long duration under standard heat-induced resin curing [31].

To the above, two other effects are added in the case of the MUF + citric acid + organic base. These are as follows:

(iv). The additional cross-linking by citric acid of some amine groups of the MUF resin [3] and of some the hydroxymethyl groups formed by formaldehyde on the melamine and/or the urea of the MUF resin.

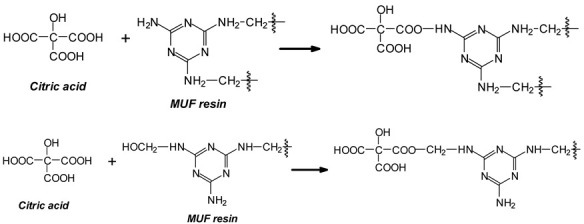


Furthermore, there is very probable covalent linking to the wood constituents of some citric acid residues already linked to the MUF.


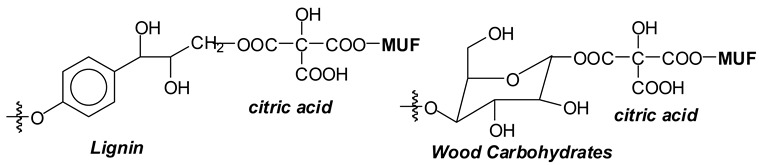


It must be considered that in the case of the hexamine, the real bases, due to its partial decomposition, are reactive imino-amine methylene bases (CH_2_=N-CH_2_^+^) [30,36,38,39,40]. This appears to be the case, as indicated by the species at 215 Da (Table S1, Figure S1 Supplementary Material). This means that some reaction of citric acid with the latter compounds cannot be excluded, but it appears from Figure S1 that their proportion is very low.

(v). The direct linking of citric acid with the carbohydrate and lignin of the wood substrate, including cross-linking through its multiplicity of carboxylic moieties of wood constituents belonging to different surfaces [3]. These reactions in which citric acid can cross-link carbohydrates and lignin units through a wood interface have already been shown to occur and reported in previous work [3].

All of this is superimposed onto the normal melamine, urea and formaldehyde condensation of the MUF resin.


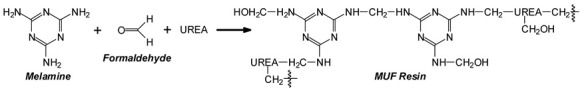


## 4. Conclusions

A self-neutralizing system to avoid damage to the constituents of wood substrates caused by too low an acidity at the wood’s interface with an adhesive where the adhesive is reinforced by extra cross-linking due to the addition of multiprotic organic acids, in particular, citric acid, has been shown to work quite effectively. The application of such a system also leads to a considerable improvement in the strength of both plywood and particleboard with, respectively, the dry and after-boiling IB strength being higher by 17% and 82% than for the control. The same trend occurs for the dry and after-hot-water strength of plywood with their strength being, respectively, 37% and 90% higher than for the control. It is worth noting that the major improvement is in the hot water resistance of the panels, and hence, in their exterior durability. The reasons for the manner in which this system works for the relatively complex MUF adhesive indicate that such an approach can be used for other adhesives as well, with some adjustments for adhesives with different characteristics. This indicates that citric acid can definitely contribute successfully with no danger to the wood interface in wood adhesive practices.

## Figures and Tables

**Table 1:** Oligomers found in the MUF + citric acid resin as determined by MALDI-ToF.

154 Da	M-CH_2_OH without Na+, (exp. 154.2 Da)
177 Da	M-CH_2_OH with Na+, (exp. 177.2 Da)
215 Da	CH_2_=N-CH_2_-OC-C(OH)(COOH)-COOH, no Na+
305 Da	HOOC-C(OH)(COOH)-CO-NH-M with Na+ (exp.301.2 Da)
	Thus CITRIC-NH-M
334 Da	HOCH_2_-M-CH_2_-M(CH_2_^+^)-CH_2_OH without Na+
357 Da	HOCH_2_-M-CH_2_-M(CH_2_^+^)-CH_2_OH with Na+
359 Da	HOOC-C(OH)(COOH)-COO-CH_2_-U-CH_2_-U with Na+ (exp. 361 Da)
	Thus, CITRIC-O-CH_2_-U-CH_2_-U
412 Da	M-NH-OC-C(OH)(COOH)-CO-NH-M with Na+, (exp.409 Da)
	Thus, M-NH-CITRIC-NH-M
533 Da	with Na+ (exp. 535–536 Da)
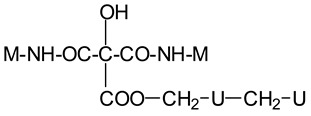	
696 Da	(exp. 697–698 Da)
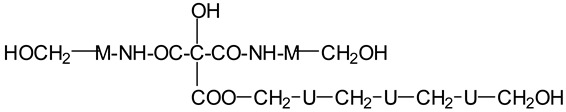	
874 Da	
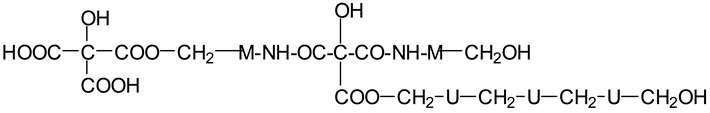	

**Table 2:** Plywood and particleboard results.

Plywood	Shear Strength Dry	Shear Strength 3 h 63 °C	
	(MPa)	(MPa)	
MUF control	0.96 + 0.07	0.20 + 0.02	
MUF + hexa/citric	1.32 + 0.09	0.38 + 0.03	
**Particleboards**	**IB strength dry**	**IB strength 2 h boiling**	**Density**
	**(MPa)**	**(MPa)**	**(kg/m^3^)**
MUF + hexa/citric	1.15	0.32	701
MUF + hexa/p-TSA	1.17	0.31	705
MUF Control	1.01	0.17	694

## Data Availability

The data are available in this article and in its Supplementary Materials.

## Supplementary Materials

The following supporting information can be downloaded at: https://www.mdpi.com/article/10.3390/polym16131819/s1, Table S1. Oligomers found in the MUF+citric acid resin as obtained by MALDI-ToF. Figure S1. MALDI ToF spectra of MUF resin+ 20% citric acid in the (a) 100 Da–800 Da range, (b) 800 Da–1000 Da range. Figure S2. MALDI ToF spectra of MUF resin alone in the (a) 20 Da–200 Da range, (b) 200 Da–400 Da range, (c) 400 Da–600 Da range, (d) 600 Da–800 Da range, (e) 800 Da–1000 Da range, (f) 1000 Da–2000 Da range.
