# Supplementary material for: Self-Neutralizing Melamine–Urea–Formaldehyde–Citric Acid Resins for Wood Panel Adhesives
Source: Polymers (Basel). 2024 Jun 27;16(13):1819. doi: 10.3390/polym16131819 (PMC11244133; doi:10.3390/polym16131819)
Supplement: Supplementary file 1 [file polymers-16-01819-s001.zip › polymers-3066187-supplementary.pdf]

SUPPLEMENTARY MATERIAL

Table S1. Oligomers found in the MUF+citric acid resin as obtained by MALDI-ToF.

154 Da	M-CH_2OH without Na+, (exp. 154.2 Da)
177 Da	M-CH_2OH with Na+, (exp. 177.2 Da)
215 Da	CH_2=N-CH_2-OC-C(OH)(COOH)-COOH, no Na+
305 Da	HOOC-C(OH)(COOH)-CO-NH-M with Na+ (exp.301.2 Da)
	Thus CITRIC-NH-M
334 Da	$HOCH_2$-M-CH_2-M(CH_2^+)-CH_2OH without Na+
357 Da	$HOCH_2$-M-CH_2-M(CH_2^+)-CH_2OH with Na+
359 Da	HOOC-C(OH)(COOH)-COO-CH_2-U-CH_2-U with Na+ (exp. 361 Da)
	Thus, CITRIC-O-CH_2-U-CH_2-U
412 Da	M-NH-OC-C(OH)(COOH)-CO-NH-M with Na+, (exp.409 Da)
	Thus, M-NH-CITRIC-NH-M
533 Da	with Na+ (exp. 535-536 Da)
	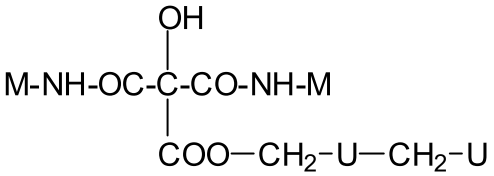
696 Da	(exp. 697-698 Da)
	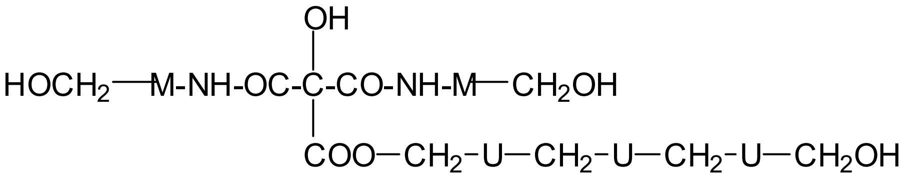
874 Da	
	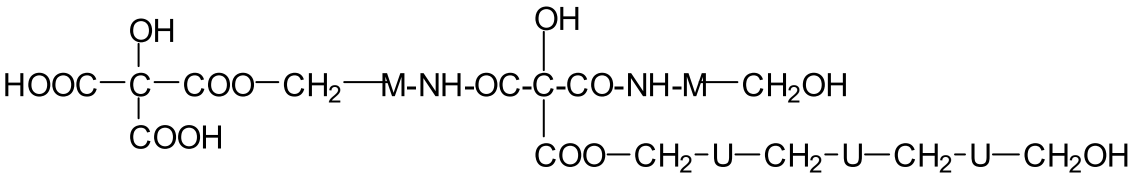

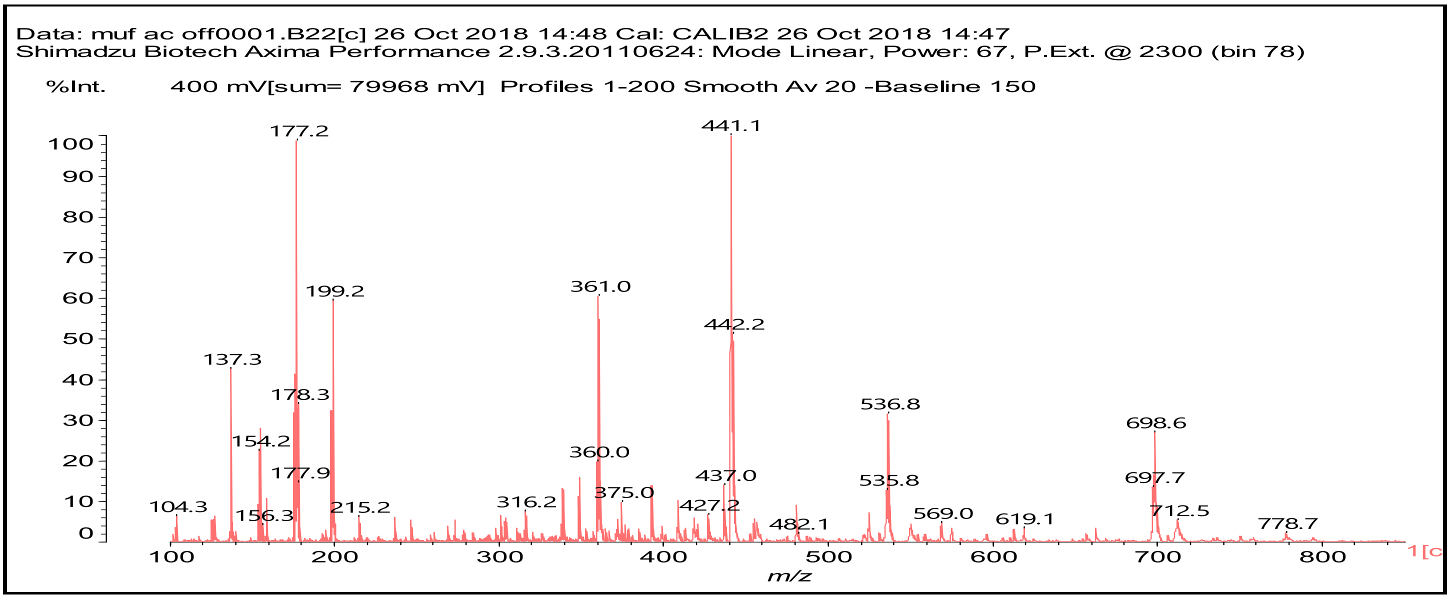

Figure S1 a

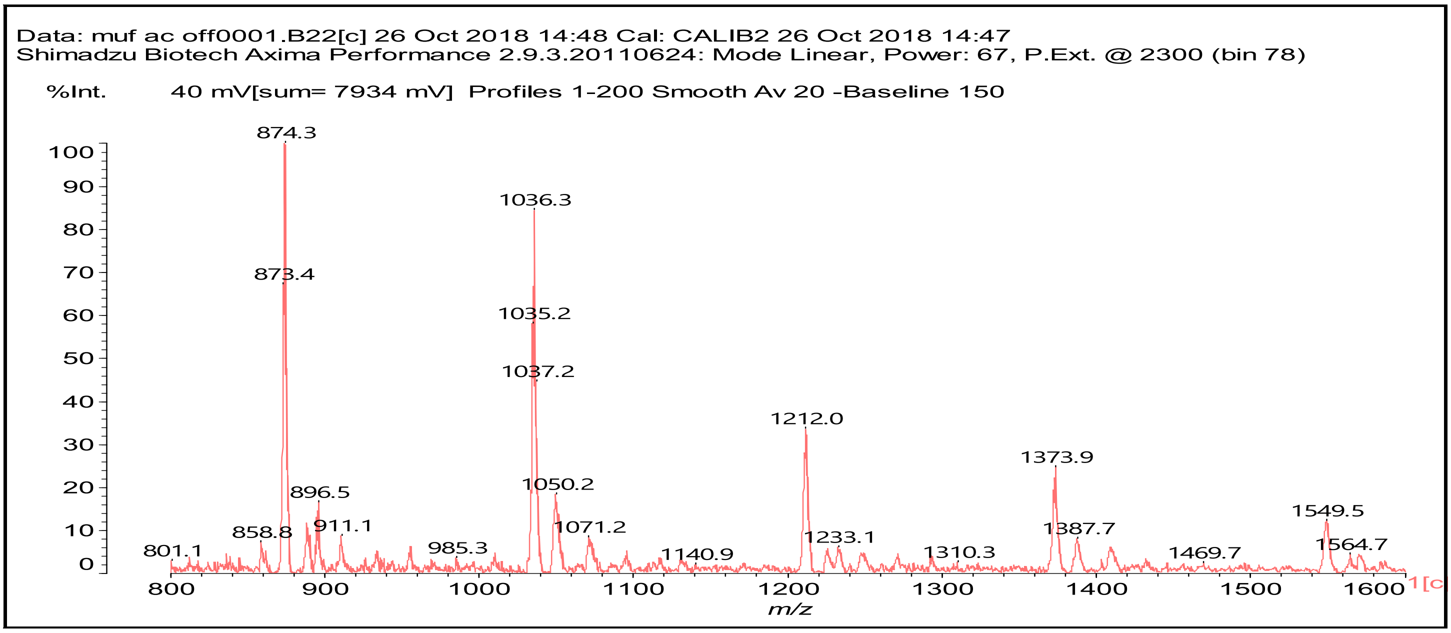

Figure S1 b.

Figure S1. MALDI ToF spectra of MUF resin+ 20% citric acid in the (a) 100 Da – 800 Da range, (b) 800 Da- 1000 Da range.

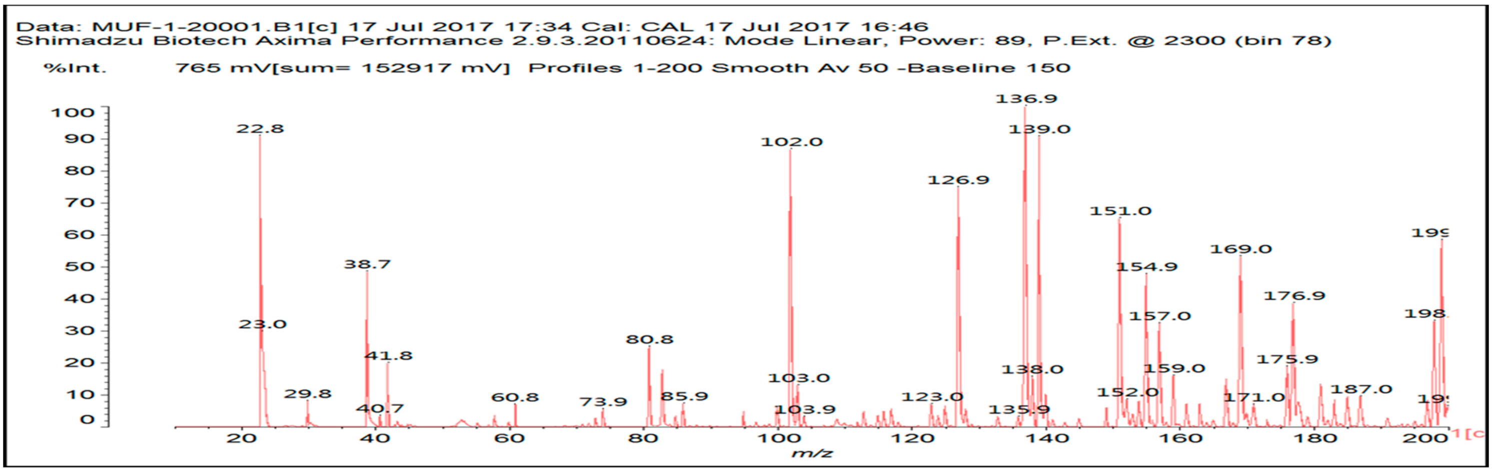
Data: MUF-1-20001.B1[c] 17 Jul 2017 17:34 Cal: CAL 17 Jul 2017 16:46
Shimadzu Biotech Axima Performance 2.9.3.20110624: Mode Linear, Power: 89, P.Ext. @ 2300 (bin 78)
%Int. 765 mV[sum= 152917 mV] Profiles 1-200 Smooth Av 50 -Baseline 150
22.8
23.0
29.8
38.7
41.8
40.7
60.8
73.9
80.8
85.9
102.0
103.0
103.9
123.0
126.9
136.9
139.0
138.0
135.9
151.0
154.9
157.0
152.0
159.0
169.0
171.0
176.9
175.9
187.0
m/z

2a

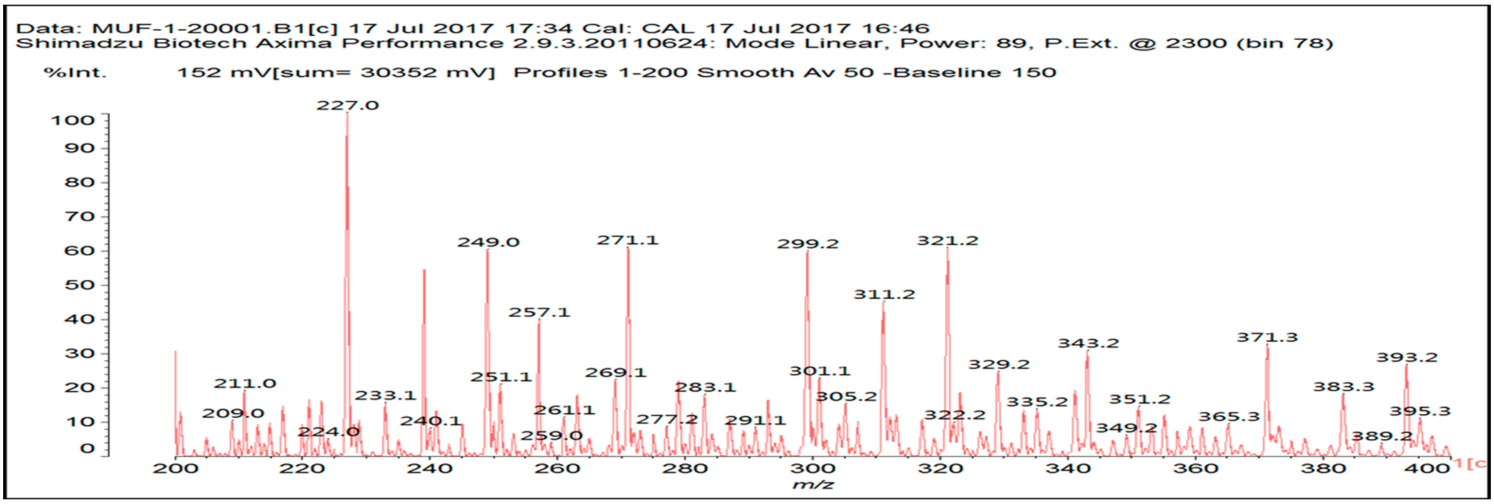
Data: MUF-1-20001.B1[c] 17 Jul 2017 17:34 Cal: CAL 17 Jul 2017 16:46
Shimadzu Biotech Axima Performance 2.9.3.20110624: Mode Linear, Power: 89, P.Ext. @ 2300 (bin 78)
%Int. 152 mV[sum= 30352 mV] Profiles 1-200 Smooth Av 50 -Baseline 150
227.0
211.0
209.0
224.0
233.1
240.1
249.0
251.1
257.1
261.1
259.0
269.1
271.1
277.2
283.1
291.1
299.2
301.1
305.2
311.2
321.2
322.2
329.2
335.2
343.2
349.2
351.2
365.3
371.3
383.3
389.2
393.2
395.3
m/z

2b

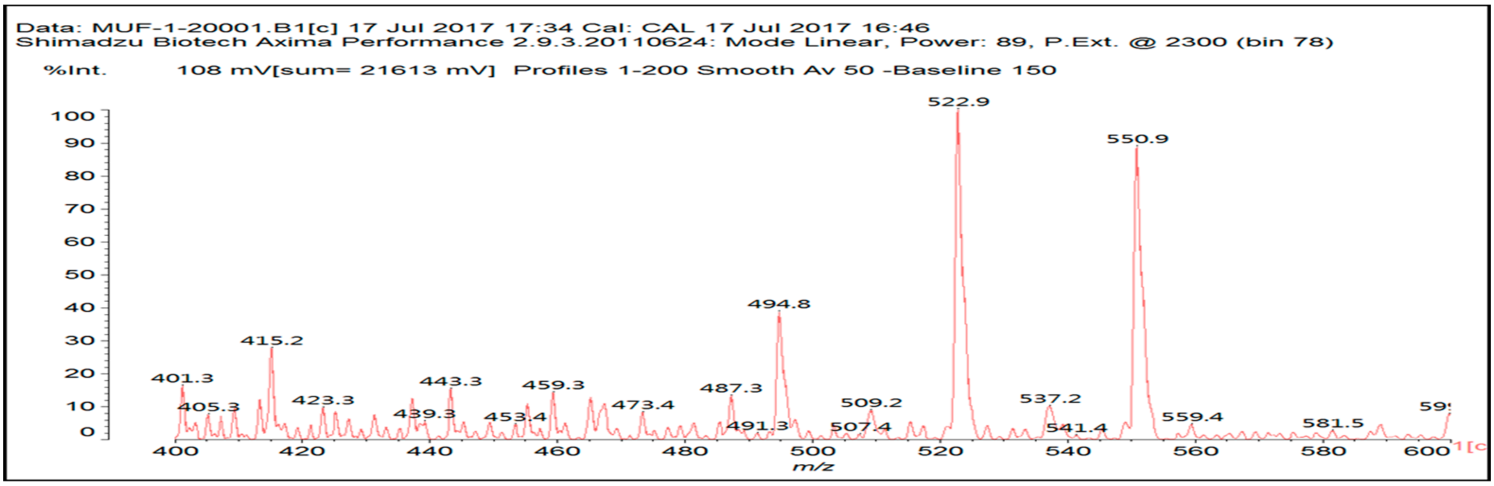
Data: MUF-1-20001.B1[c] 17 Jul 2017 17:34 Cal: CAL 17 Jul 2017 16:46
Shimadzu Biotech Axima Performance 2.9.3.20110624: Mode Linear, Power: 89, P.Ext. @ 2300 (bin 78)
%Int. 108 mV[sum= 21613 mV] Profiles 1-200 Smooth Av 50 -Baseline 150
401.3
405.3
415.2
423.3
439.3
443.3
453.4
459.3
473.4
487.3
491.3
494.8
507.4
509.2
522.9
537.2
541.4
550.9
559.4
581.5
m/z

2 c

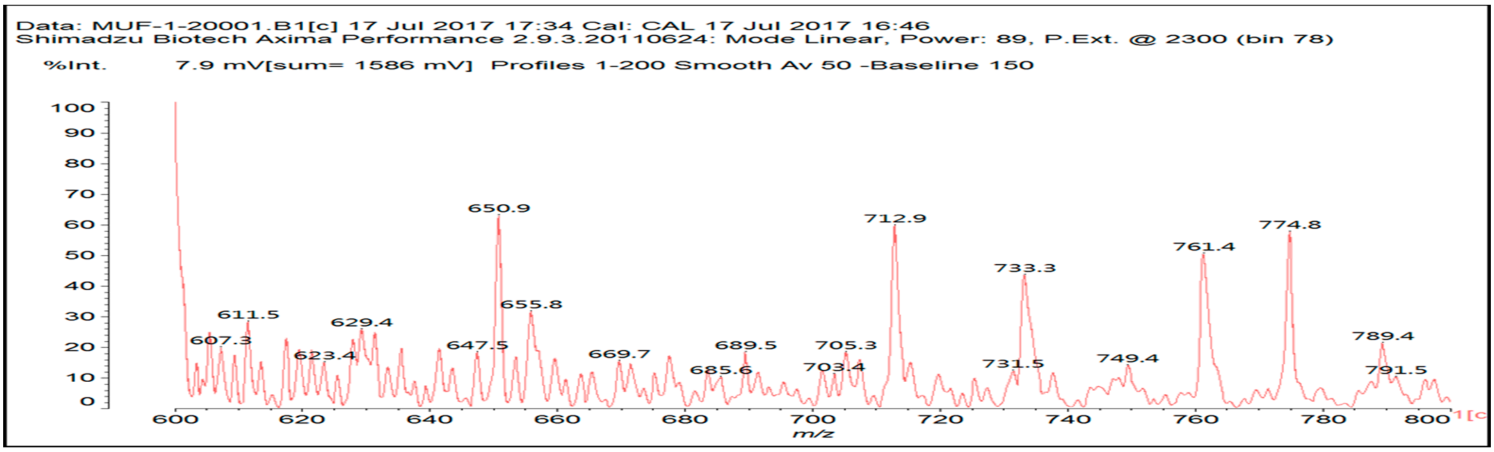

2d

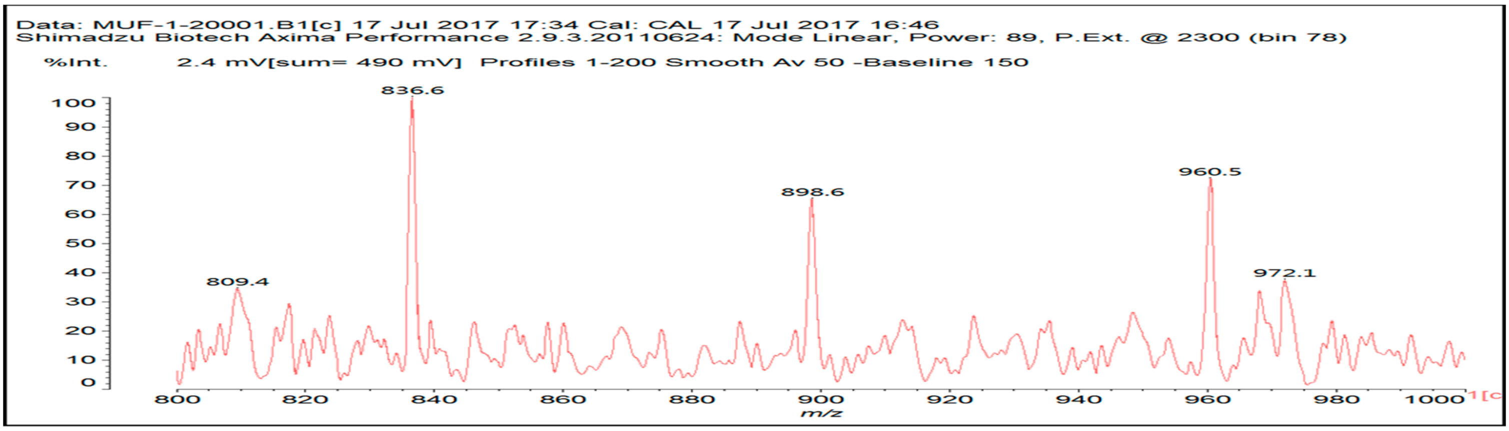

2e

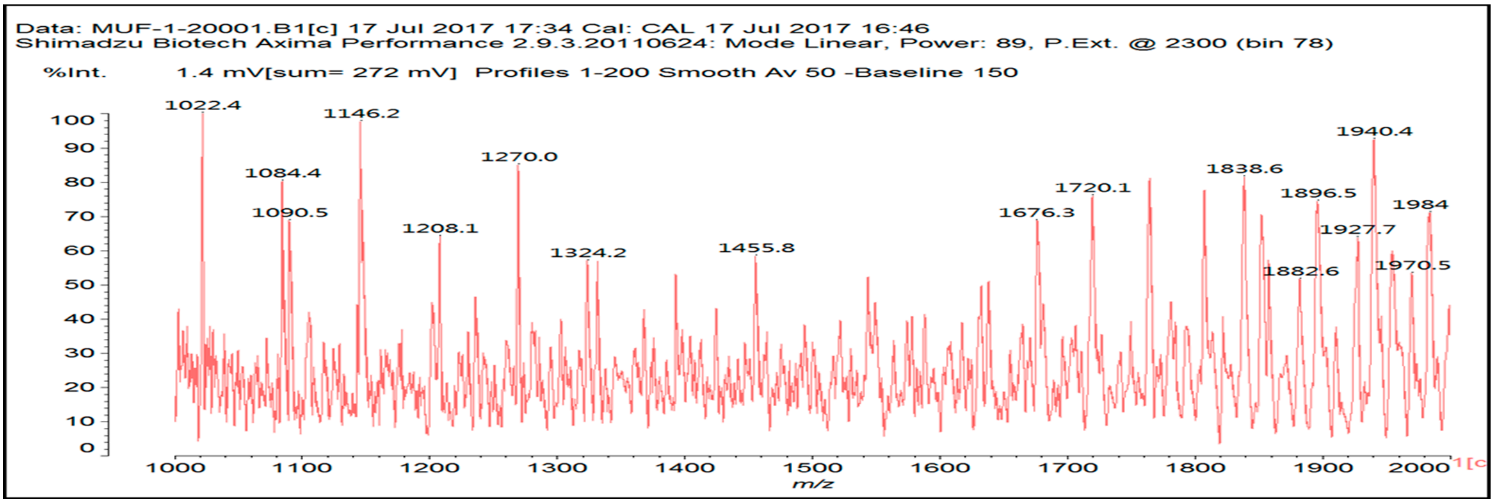

2f

Figure S2. MALDI ToF spectra of MUF resin alone in the (a) 20 Da – 200 Da range, (b) 200 Da -400 Da range, (c) 400 Da – 600 Da range, (d) 600 Da – 800 Da range, (e) 800 Da – 1000 Da range, (f) 1000 Da – 2000 Da range
